# Retraction: Maresin-1 and Resolvin E1 promote regenerative properties of periodontal ligament stem cells under inflammatory conditions

**DOI:** 10.3389/fimmu.2026.1961138

**Published:** 2026-08-13

**Authors:** Frontiers Editorial Office

The journal retracts the 25 September 2020 article cited above.

Following publication, the authors contacted Frontiers raising concerns regarding the integrity of the images in the published figures. Specifically, it was identified a duplication in Figure 3C. The authors refer to the impossibility to provide the raw data during the investigation, which was conducted in accordance with Frontiers’ policies. As a result, the data and conclusions of the article have been deemed unreliable, and the article has been retracted.

This retraction was approved by the Chief Editors of Frontiers in Immunology and the Chief Executive Editor of Frontiers. The authors agreed regarding this retraction.

